# Profiles of apple allergen components and its diagnostic value in Northern China

**DOI:** 10.3389/fmed.2024.1388766

**Published:** 2024-06-13

**Authors:** Xiaoyan Wang, Lijia Chen, Tianfei Lan, Hongtian Wang, Xueyan Wang

**Affiliations:** ^1^Department of Allergy, Beijing Shijitan Hospital, Capital Medical University, Beijing, China; ^2^Beijing Laboratory of Allergic Diseases, Beijing Municipal Education Commission, Beijing, China

**Keywords:** apple allergy, mal d 1, mal d 3, bet v 1, pollen food allergy syndrome

## Abstract

**Background:**

Limited is known on the profiles of apple allergy in China.

**Objective:**

To explore the clinical significance of apple allergen components in northern China.

**Methods:**

This study recruited 40 participants and categorized into apple tolerance (*n* = 19) and allergy (*n* = 21) group. The latter was categorized into oral allergy symptoms (OAS, *n* = 14) and generalized symptoms (GS, *n* = 7). All participants underwent ImmunoCAP screening to assess sIgE levels of birch, apple, and their components.

**Results:**

The sensitization rates were 90% for Bet v 1, 85% for Mal d 1, 35% for Bet v 2, and 20% for Mal d 3. The overall positive rate for apple allergens was 97.5%, with half demonstrating mono-sensitization to Mal d 1. Birch, Bet v 1 and Mal d 1 sIgE levels had consistent areas under the curve (AUC 0.747, *p* = 0.037; AUC 0.799, *p* = 0.012; AUC 0.902, *p* < 0.001 respectively) in diagnosing apple allergy. The optimal cut-off values were determined to be 22.85 kUA/L (63.6% sensitivity, 85.7% specificity), 6.84 kUA/L (81.8% sensitivity, 71.4% specificity) and 1.61 kUA/L (93.8% sensitivity, 75.0% specificity), respectively. No allergens or components demonstrated diagnostic value in distinguishing between OAS and GS. Mal d 3 sensitization was correlated with mugwort allergy and higher risk of peach, nuts or legumes generalized allergy.

**Conclusion:**

Mal d 1 was major allergen and the best for diagnosing apple allergy. Mal d 3 does not necessarily indicate severe allergic reaction to apples in northern China but may indicate mugwort sensitization and an increased risk of peach, nuts or legumes allergy.

## Graphical abstract

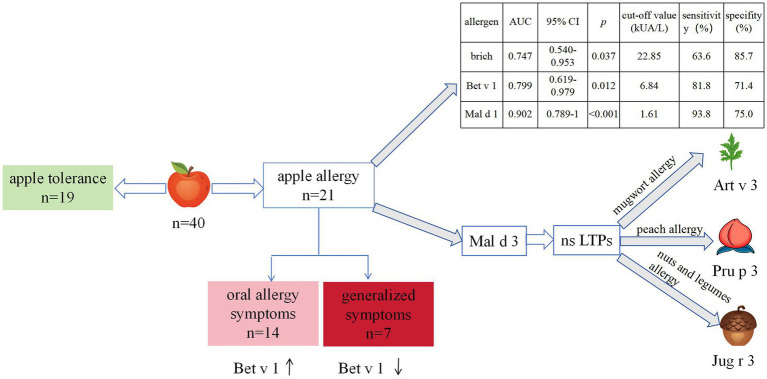

## 1 Introduction

Food allergy has garnered increasing global attention in recent years (1–3). The prevalence of fruit and vegetable allergies has been on the rise among commonly encountered food allergens (2–4). This condition is known as pollen food allergy syndrome (PFAS), a prevalent allergic disorder characterized by cross-reactivity between pollens and plant-derived substances (5, 6). Within the PFAS group, *Rosaceae* species such as *Amygdaleae* (almond/peach/cherry/apricot/plum), *Maleae* (apple/pear), and *Rosoideae* (raspberry/blackberry/strawberry) are prominent food allergens (7).

Apple species (*Malus domestica*) is the third most cultivated fruit in the world. In addition to its nutritional richness, apple has emerged as a prominent global allergen for PFAS, contributing significantly to allergic reactions worldwide (5, 8). Apple is identified as the predominant food allergen, particularly in Central-Northern Europe (2, 9). In Korea and Japan, apple allergy is still the leading food allergy (10–12). According to our unpublished data on the prevalence of PFAS in northern China, apple ranked third. This condition was particularly evident in patients with birch pollen allergy (13–15). Symptoms induced by apple vary across Europe, ranging from mild local symptoms to generalized symptoms, even anaphylaxis, with variations observed between northern and southern regions (16–18). Severe symptoms of apple allergy was not common in birch endemic regions in north Europe. 2.5% of the apple allergy patients suffered from severe symptoms in Netherlands (19), while it was more than 50% in Mediterranean area (16).

With the advent of allergen component resolved diagnosis (CRD), the elucidation of apple allergy’s molecular basis among PFAS has been achieved. To date, the World Health Organization/International Union of Immunological Societies (WHO/IUIS) has officially recognized and incorporated four allergens into the nomenclature for apples, namely Mal d 1, Mal d 2, Mal d 3, and Mal d 4 (20). These allergens include pathogenesis-related class 10 proteins (PR-10s), thaumatin-like proteins (TLPs), non-specific lipid transfer proteins (nsLTPs), and profilins, which are associated with PFAS and LTP allergies (21). The allergen components sensitization profile of apple was previously explored across Europe and Japan (12, 13, 18, 19, 22, 23). The sensitization rates of Mal d 1 and Mal d 3 exhibited a positive correlation with the severity disparity of symptoms, thereby indicating a regional variation. Therefore, a comprehensive investigation into the local sensitization characteristics of apple components could facilitate the prediction of allergic symptoms and enhance the management of affected patients.

The clinical significance of apple allergen components remains uncertain, particularly in northern China characterized by high levels of birch pollen. Only one study conducted in northern China, involving 28 participants, reported the sensitization rate of Mal d 1; However, the study did not encompass an analysis of other components and offer a comprehensive explication of its clinical significance (24). In previous study, we have reported a higher level of Bet v 1 sIgE in PFAS and apple allergic patients compared to those without food allergy (15). The objective of this study was to elucidate the clinical efficacy of CRD in Chinese patients with apple allergy coexisting with birch pollen allergy, and to investigate the differential diagnostic value of Mal d 1 and Mal d 3.

## 2 Methods

### 2.1 Study population

A cross-sectional study was conducted, enrolling 40 patients with birch pollen allergy who were admitted to the Department of Allergy at Beijing Shijitan Hospital from March 2022 to April 2023. All 40 participants underwent apple allergen screening via either a skin prick test or serum sIgE level test, yielding positive results.

### 2.2 Questionnaire

Under the guidance of an allergy specialist, patients or their legal guardians were asked to complete a questionnaire regarding their social demographics, clinical history, comorbidities. Furthermore, PFAS questionnaire including the culprit foods (fruits, vegetables, legumes, grains, nuts and others), type of symptoms was finished. The clinical evaluation included a comprehensive medical history, encompassing detailed information on allergies to pollen and plant-based food.

### 2.3 Apple allergy classification

Based on a robust history of immediate acute allergic reactions upon apple ingestion and/or positive outcomes from open food challenges, the subjects were categorized into two groups: those with apple tolerance and those with apple allergy (25). All subjects received an open apple challenge test adapted from references (26, 27). The apples (cultivar *Hongfushi* with medium size) were obtained from the same market and stored under identical conditions. Initially, patients consumed 10 g of unpeeled apple. Subsequent doses were increased every 20 min, starting with 20 g, then 40 g, and finally reaching 80 g. The challenge ended when subjects ingested 80 g of apple or experienced cutaneous and/or respiratory symptoms or changes in vital signs.

Among the apple allergy group, patients were classified into two groups based on symptom severity: the oral allergy symptoms (OAS) group, characterized by oral mucosal symptoms; and the generalized symptoms (GS) group, presenting with systemic manifestations such as generalized urticaria, angioedema, laryngeal edema, respiratory distress, gastrointestinal disorders or circulatory collapse indicative of anaphylaxis (25).

### 2.4 Ethics statement

Each participant or their legal guardian provided written informed consent, and the Ethics Committee of Beijing Shijitan Hospital, Capital Medical University granted approval for this study (No. 2022–081).

### 2.5 Allergen screen of pollen and food allergy

All participants underwent screening for sIgE levels of birch, mugwort pollen and apple allergen, as well as total IgE levels using ImmunoCAP (ThermoFisher Scientific, Uppsala, Sweden). Meanwhile, ImmunoCAP analysis was performed to assess the allergen components of birch (Bet v 1, Bet v 2, Bet v 4, and Bet v 6) and apple (Mal d 1, Mal d 3). A positive outcome was defined as sIgE levels exceeding 0.35 kUA/L.

A skin prick test was conducted on the flexor surface of the forearm by using the prick-to-prick-technique (28). Histamine dihydrochloride (10 mg/mL) was used as a positive control. Apples (cultivar *Hongfushi* with medium size) were gathered from the same market and stored under the same conditions. Tests were performed using the pulp of the apple obtained from the middle area of the fruit. The wheal reaction was measured after 15 min and the presence of a wheal with a diameter exceeding 3 mm was considered as indicative of a positive result.

### 2.6 Statistics analyses

Statistical analysis of the data was conducted using SPSS 25.0 software package (IBM SPSS Statistics, Armonk, NY) and Prism 8.0 software (GraphPad Software Inc., San Diego, CA, United States). Categorical data were presented as frequencies (n) and proportions (%), while quantitative data were analyzed using either mean and standard deviation (SD) or median and interquartile range (IQR). The independent group *t* tests were used for comparing normally distributed continuous variables, whereas Mann–Whitney U tests were used for comparing non-normally distributed variables. The chi-square test was used to compare proportions. Spearman correlation analysis was conducted to evaluate the relevance. Receiver operating characteristics (ROC) curves were used to assess the diagnostic value of the sIgE levels of allergen and its components. Differences with a *p* < 0.05 were considered significant.

## 3 Results

### 3.1 Clinical demographics of studied subjects

The study included a total of 40 patients who were both sensitized to birch pollen and apple. The median age was 30.0 (IQR: 24.0, 38.8) years old and 21 (52.5%) were female. Of these, 8 (20.0%) were children. Among them, 8 (20%) individuals reported only spring pollen allergy symptoms while 32 (80%) individuals experienced both spring and autumn symptoms. Additionally, 12 cases (30%) were combined with asthma while the remaining 28 (70%) only had AR (Table 1).

**Table 1:** Comparison of demographic clinical characteristics of apple allergy subjects between apple tolerance and apple allergy group.

Variants	Total (*n* = 40)	Apple tolerance (*n* = 19)	Apple allergy (*n* = 21)	p
Age (y), media (IQR)	30.0 (24.0, 38.8)	32 (16, 40)	29 (26, 38)	0.86
Age group, *n* (%)				0.342
Adult	32 (80.0)	14 (73.7)	18 (85.7)	
children	8 (20.0)	5 (26.3)	3 (14.3)	
Gender, male, *n* (%)	19 (47.5)	9 (47.4)	10 (47.6)	0.987
Seasonality, *n* (%)				0.527
Spring	8 (20.0)	3 (15.8)	5 (23.8)	
Spring and autumn	32 (80.0)	16 (84.2)	16 (76.2)	
Combined diseases, *n* (%)				0.836
Only AR	28 (70.0)	13(68.4)	15 (71.4)	
AR + asthma	12 (30.0)	6 (31.6)	6 (28.6)	
Allergic to peach, *n* (%)	22 (55.0)	4 (21.1)	18 (85.7)	<0.001
Allergic to nuts and legumes, *n* (%)	19 (47.5)	8 (42.1)	11 (52.4)	0.516
Allergen positive rate, *n* (%)				
Birch	40 (100)	19 (100)	21 (100)	1
Apple	28 (70.0)	11 (57.9)	17 (81.0)	0.112
Mugwort	27 (67.5)	14 (73.7)	13 (61.9)	0.427
Allergen component positive rate, *n* (%)				
Bet v 1	36 (90.0)	16 (84.2)	20 (95.2)	0.246
Bet v 2	14 (35.0)	9 (47.4)	5 (23.8)	0.119
Bet v 4	0 (0)	0 (0)	0 (0)	NA
Bet v 6	0 (0)	0 (0)	0 (0)	NA
Mal d 1	34 (85.0)	14 (73.7)	20 (95.2)	0.057
Mal d 3	8 (20.0)	5 (26.3)	3 (14.3)	0.342
Positive apple components, *n* (%)				0.462
0	1 (2.5)	1 (5.3)	0 (0)	
1	24 (60.0)	10 (52.6)	14 (66.7)	
2	14 (35.0)	7 (36.8)	7 (33.3)	
3	1 (2.5)	1 (5.3)	0 (0)	

NA, not available; AR, allergic rhinitis.

Out of the 40 subjects, 19 cases (47.5%) belonged to the apple tolerance group, while the remaining 21 cases (52.5%) were classified as the apple allergy group. Additionally, the apple allergy group was further categorized into OAS group (*n* = 14, 66.7%) and GS group (*n* = 7, 33.3%) (Table 1). Among those with GS, 6/7 were suffered from cutaneous symptoms, 4/7 suffered from respiratory symptoms and only 1/7 presented with gastrointestinal symptoms. None of them suffered from severe reactions of apple allergy including anaphylaxis (Table 2). The incidence of seasonality or combined disease did not differ significantly between the apple allergy and tolerance group.

**Table 2:** Allergic symptoms triggered by apple.

Symptoms	n	%
Without symptoms	19	47.5
Localized oropharyngeal	14	35.0
Generalized	7	17.5
Cutaneous	6	15.0
Respiratory	4	10.0
Cardiovascular	0	0
Gastrointestinal	1	2.5
Neurological	0	0
Anaphylaxis	0	0

Among enrolled subjects, 22 (55.0%) were allergic to peach while 19 (47.5%) were allergic to nuts and legumes. Subjects with apple allergy were found to have a higher risk of peach allergy compared with apple tolerance subjects (85.7% vs. 21.1%, *p* < 0.001; Table 1). All 7 cases from the GS group exhibited peach allergy, while 4 out of 7 also demonstrated nut and legumes allergy (Table 3). GS group tends to present spring and autumn allergic symptoms compared with OAS group although no significant differences were observed (*p* = 0.07; Table 3).

**Table 3:** Comparison of demographic clinical characteristics of apple allergy subjects between apple tolerance and apple allergy group.

Variants	OAS group (*n* = 14)	GS group (*n* = 7)	p
Age (y), media (IQR)	29 (22.5, 35)	30 (27, 38)	0.575
Age group, *n* (%)			0.186
Adult	11 (78.6)	7 (100)	
Children	3 (21.4)	0 (0)	
Gender, male, *n* (%)	7 (50.0)	2 (57.1)	0.757
Seasonality, *n* (%)			0.07
Spring	5 (35.7)	0 (0)	
Spring and autumn	9 (64.3)	7 (100)	
Combined diseases, *n* (%)			0.306
Only AR	11 (78.6)	4 (71.4)	
AR + asthma	3 (21.4)	3 (28.6)	
Allergic to peach, *n* (%)	11 (78.6)	7 (100)	0.186
Allergic to nuts and legumes, *n* (%)	7 (50.0)	4 (57.1)	0.757
Allergen positive rate, *n* (%)			
Birch	14 (100)	7 (100)	1
Apple	11 (78.6)	6 (85.7)	0.694
Mugwort	8 (57.1)	5 (71.4)	0.525
Allergen component positive rate, *n* (%)			
Bet v 1	14 (100)	6 (85.7)	0.147
Bet v 2	4 (28.6)	1 (14.3)	0.469
Mal d 1	14 (100)	6 (85.7)	0.147
Mal d 3	2 (14.3)	1 (14.3)	1
Numbers of apple allergen components, *n* (%)			0.19
1	8 (57.1)	6 (85.7)	
2	6 (42.9)	1 (14.3)	

AR, allergic rhinitis; GS, generalized symptoms; OAS, oral allergy symptoms.

### 3.2 Sensitization levels and patterns of allergen and Its components

**Figure 1:**
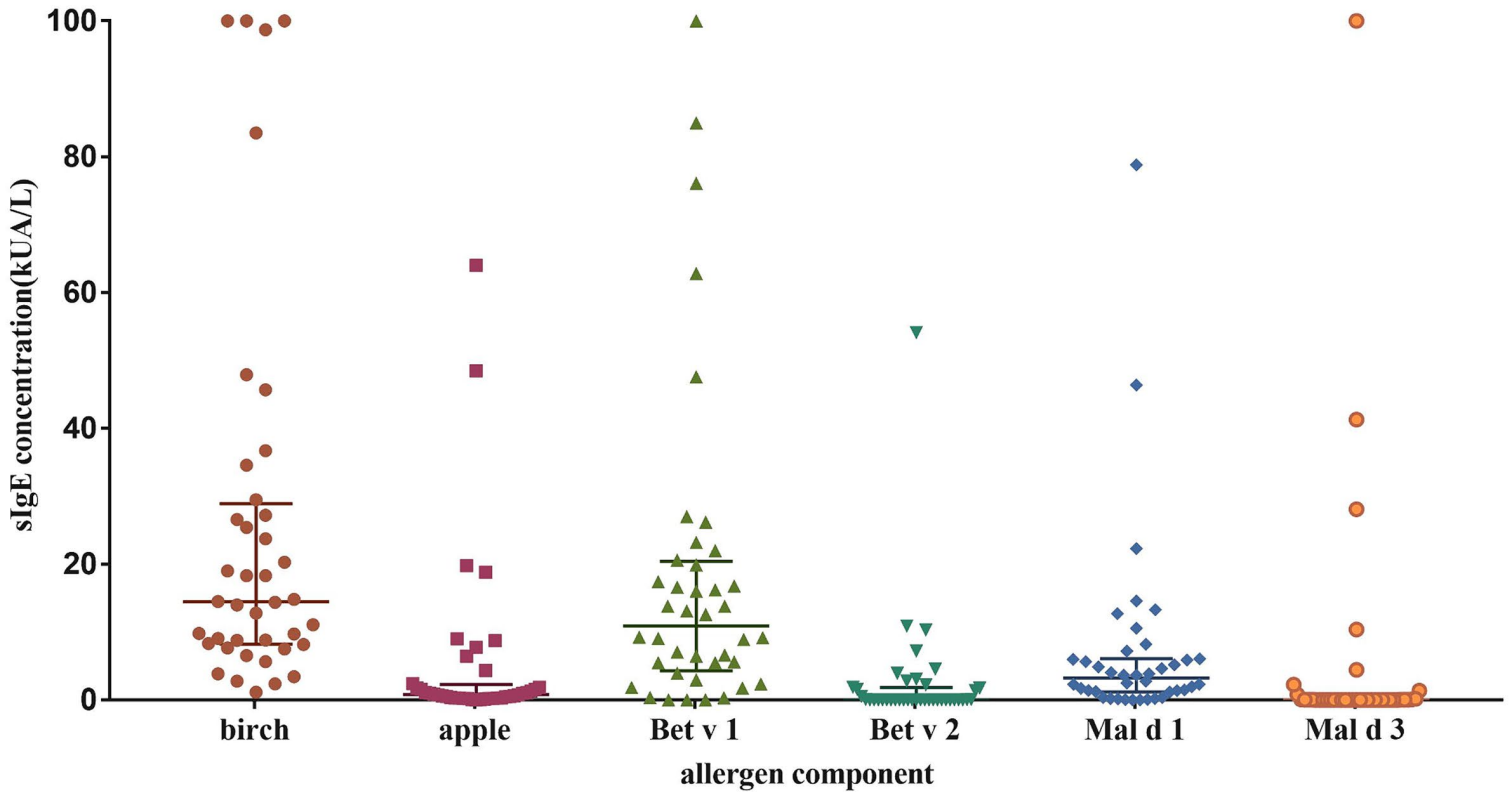
Levels of sIgE against different allergens and their components.

**Figure 2:**
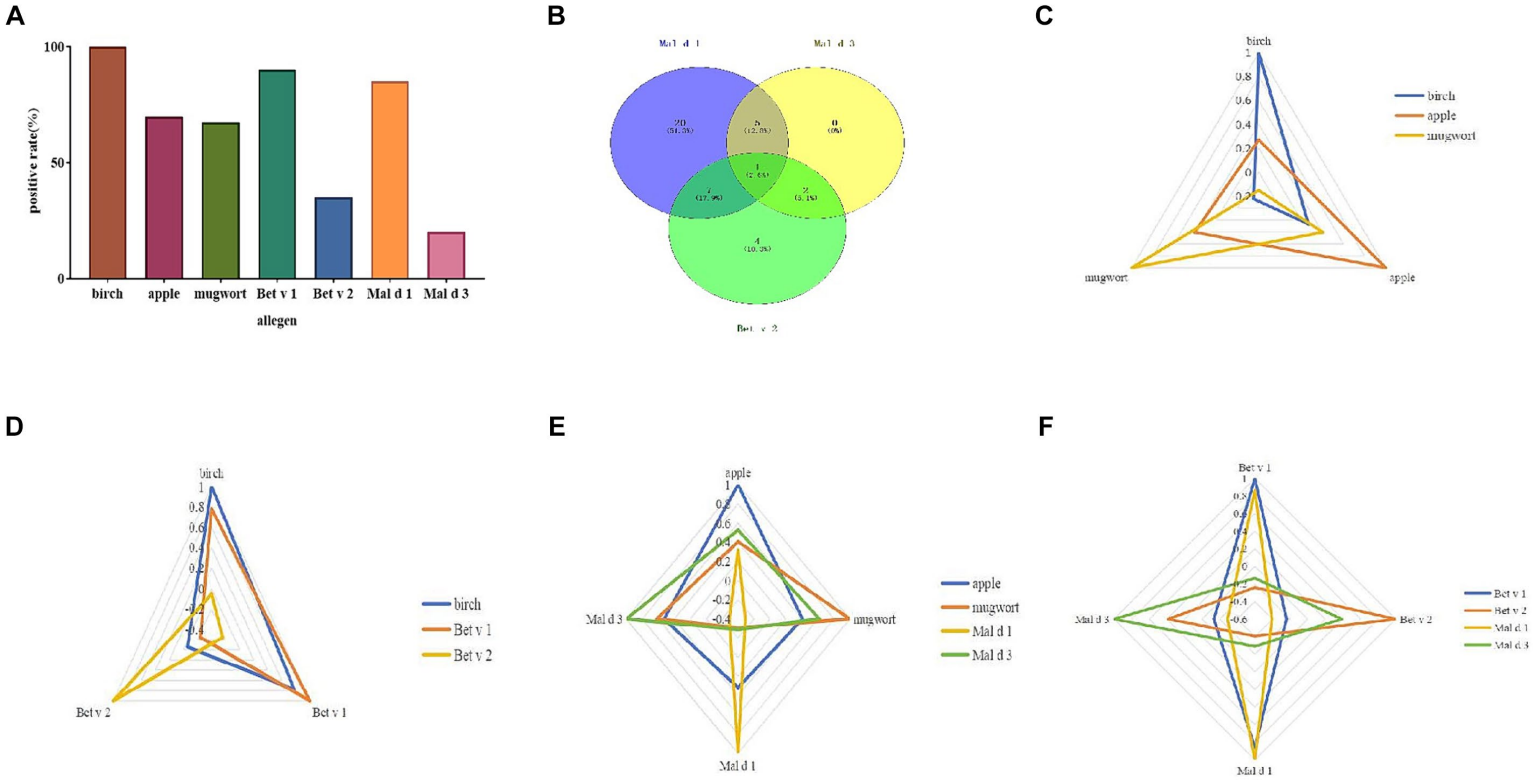
Allergen sensitization pattern. The positive rate of allergen and its components **(A)**. Venn diagram of three apple allergen components **(B)**. Correlation analysis of different allergens and their components by radar map **(C–F)**.

The sIgE levels was shown in Figure 1. The highest level was seen in birch (median 14.5 kUA/L; IQR 8.2–28.9 kUA/L), followed by Bet v 1 (median 10.9 kUA/L; IQR 4.3–20.4 kUA/L) and Mal d 1 (median 3.2 kUA/L; IQR 1.2–6.1 kUA/L). The sensitization rate for birch was 100%, while it was 70% for apple, 67.5% for mugwort. As regarding to allergen components, the positive rate was 90% for Bet v 1, 85% for Mal d 1, 35% for Bet v 2 and 20% for Mal d 3 (Figure 2A).

The sensitization pattern of apple components was analyzed by considering Bet v 2 (profilin) as a substitute for Mal d 4. The overall positive rate to at least one allergen component of apple was 97.5%. Only 1 case (2.5%) showed a sIgE level of 0.16 kUA/L for Mal d 1. The apple allergen elicited mono-sensitization in 60% of the patients, while dual sensitization was observed in 35% of the subjects. Only one patient (2.5%) was sensitized to all three components (Figure 2B). Half of the patients exhibited mono-sensitization to Mal d 1, while 4 cases (10%) demonstrated mono-sensitization to Bet v 2. Among the 4 patients, only one (25%) belonged to OAS group, while the remaining three were classified as tolerance group. On the contrary, 13/20 (65%) of Mal d 1 mono-sensitized subjects suffered from allergic symptoms to apple. 5/13 (38.5%) of them suffered from generalized symptoms of apple allergy. No significant difference of prevalence of apple allergy was found between mono and double sensitized components (Table 1).

Mal d 1 displayed positive correlations with birch (*p* < 0.001), apple (*p* = 0.046), Bet v 1 (*p* < 0.001), while exhibiting negative correlations with Bet v 2 (*p* = 0.009) and Mal d 3 (*p* = 0.07; Figures 2C–F).

### 3.3 Differences of allergen sensitization between apple allergy and tolerance group

The sensitization rate was higher in apple allergy group compared with tolerance group for sIgE levels of Bet v 1 and Mal d 1 but with no significant differences (*p* = 0.246, *p* = 0.057, respectively; Table 1).

**Figure 3:**
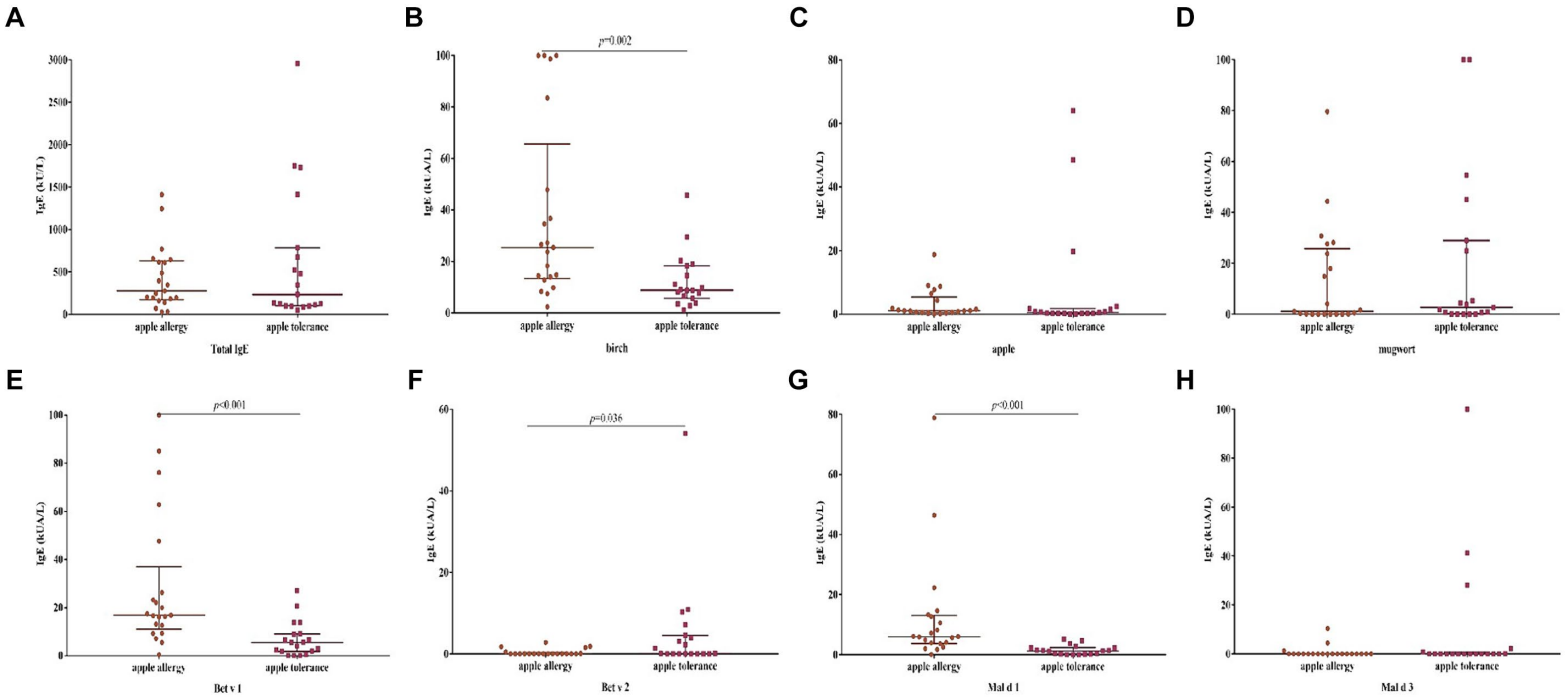
Differences of levels against allergen sIgE between apple allergy and apple tolerance group. Significant differences were observed in birch (*p* = 0.002) **(B)**, Bet v 1 (*p* < 0.001) **(E)**, Bet v 2 (*p* = 0.036) **(F)** and Mal d 1 (*p* < 0.001) **(G)**. No significant differences were found between two groups in total IgE **(A)**, apple **(C)**, mugwort **(D)** and Mal d 3 **(H)**.

The levels of sIgE against birch, Be v 1 and Mal d 1 were significantly elevated in apple allergy group compared to tolerance group (*p* = 0.002, *p* < 0.001, *p* < 0.001, respectively; Figure 3). Interestingly, level of Bet v 2 sIgE was conversely higher in apple tolerance group than apple allergy group (*p* = 0.036). The levels of apple, mugwort, Bet v 4, and Mal d 3 were comparable in both groups without any significant differences.

The level of Bet v 1 sIgE was significantly higher in OAS group compared to GS group (*p* = 0.037; Table 4), while no significant differences were found against levels of other allergens. The positive rate of allergen components did not exhibit any significant differences between the apple allergy and tolerance groups, as well as the OAS and GS group (Table 3).

**Table 4:** Differences of allergen sIgE levels between OAS and GS subgroup in apple allergy subjects (kUA/L).

Allergen sIgE level median (IQR)	OAS group (*n* = 14)	GS group (*n* = 7)	p
Birch	30.9 (13.5, 87.3)	14.4 (12.8, 25.4)	0.204
Apple	1.2 (0.5, 5.5)	0.8 (0.5, 6.5)	0.456
mugwort	0.8 (0, 20.6)	4.1 (0.1, 27.7)	0.473
Bet v 1	21.0 (15.7, 54.7)	12.6 (7.1, 16.2)	0.037
Bet v 2	0 (0, 0.8)	0 (0, 0)	0.49
Mal d 1	6.0 (4.0, 16.5)	5.7 (2.5, 8.2)	0.263
Mal d 3	0 (0, 0.1)	0 (0, 0)	0.22

GS, generalized symptoms; OAS, oral allergy symptoms.

### 3.4 The diagnostic value of different allergen components

**Figure 4:**
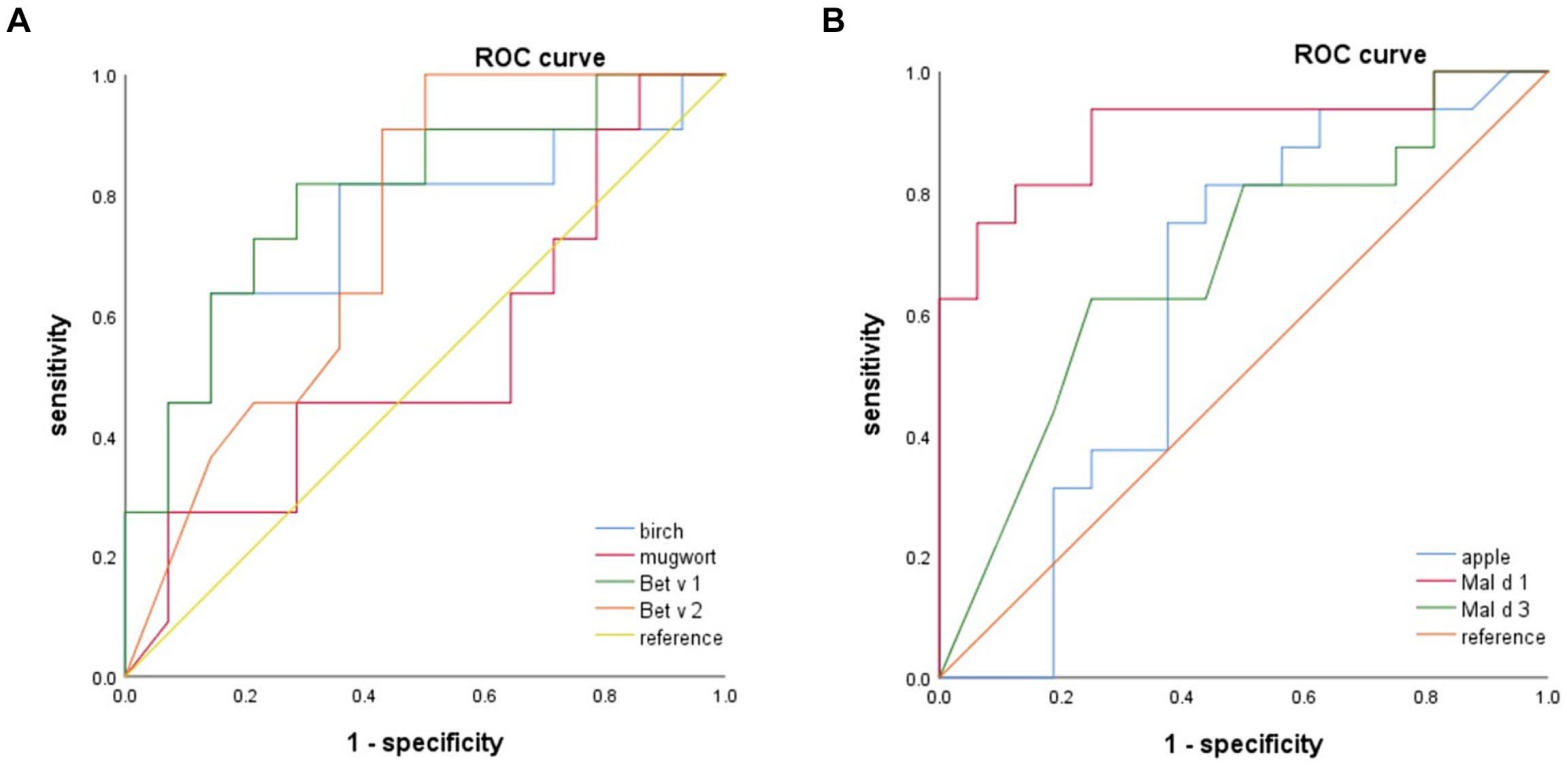
The ROC analysis indicate that birch, Bet v 1 and Mal d 1 can serve as reliable predictors for apple allergy. Mal d 1 yields the highest AUC. ROC, receiver operating curve; AUC, areas under the curve.

The ROC curve was employed to determine the optimal cutoff value of sIgE levels for discriminating between patients with apple tolerance and those with apple allergy. Birch, Bet v 1, Mal d 1, but not Mal d 3 or Bet v 2 could discriminate apple allergy from tolerance as depicted in Figure 4. ROC analysis revealed that birch, Bet v 1 and Mal d 1 sIgE levels had consistent areas under the curve (AUC 0.747, 95% CI: 0.540–0.953, *p* = 0.037; AUC 0.799, 95% CI: 0.619–0.979, *p* = 0.012; AUC 0.902, 95% CI: 0.789–1, *p* < 0.001 respectively) in diagnosing apple allergy.

The optimal cut-off values for birch, Bet v 1 and Mal d 1 sIgE were determined to be 22.85 kUA/L (63.6% sensitivity, 85.7% specificity), 6.84 kUA/L (81.8% sensitivity, 71.4% specificity) and 1.61 kUA/L (93.8% sensitivity, 75.0% specificity) respectively. The AUC of mugwort, apple, Bet v 2 and Mal d 3 was found to be insignificant when diagnosing apple allergy.

No allergen or components had diagnostic value of discriminating OAS and GS by ROC analysis.

### 3.5 The clinical significance of mal d 3

In this study, eight subjects were sensitized to Mal d 3; however, our findings indicate that Mal d 3 does not possess any diagnostic value for either apple allergy diagnosis or discrimination of allergy severity. Out of the total cases, only three individuals exhibited symptoms of apple allergy, while the remaining five cases demonstrated allergies to peach, nuts, or legumes as indicated in Table 5. All eight cases were sensitized to mugwort and allergic to nuts and legumes.

**Table 5:** Food allergy and clinical symptoms related with Mal d 3 sensitization.

		Allergen sIgE levels (kUA/L)							Food allergy		
Patients	Gender, age	Mal d 3	Birch	Apple	Mugwort	Bet v 1	Bet v 2	Mal d 1	Apple	Peach	Nuts and legumes
Patient 1	F, 14y	100	2.79	64	100	8.93	0.09	2.31	Tolerance	Tolerance	GS
Patient 2	F, 26y	41.3	6.55	19.8	45.1	2.99	0	0.38	Tolerance	GS	GS
Patient 3	M, 9y	28.1	1.13	48.5	100	0.02	3.1	0.03	Tolerance	Tolerance	GS
Patient 4	F, 39y	10.4	14.4	0.99	23.8	7.06	0.01	2.52	GS	GS	GS
Patient 5	M, 30y	4.45	8.8	2.43	29	6.61	1.36	1.41	Tolerance	GS	GS
Patient 6	M, 28y	2.26	26.6	18.8	30.7	22	0.01	13.3	OAS	OAS	OAS
Patient 7	M, 22y	1.36	20.3	1.59	3.91	0.36	10.3	0.23	Tolerance	OAS	OAS
Patient 8	M, 17y	0.79	14.8	64	44.4	13.1	0.01	1.74	OAS	Tolerance	OAS

GS, generalized symptoms; OAS, oral allergy symptoms.

## 4 Discussion

The present study unveiled the major allergen components of apple and investigated their correlation with clinical symptoms. To date, this study represents one of the few investigations into the clinical significance of different apple allergen components (Mal d 1, Mal d 3) in China. We identified a distinctive characteristic of apple allergy, resembling that observed in northern Europe but diverging from southern Europe.

Food allergy due to birch pollen related cross-reactivity was common in Central-Northern Europe as well as apple allergy (2, 18, 29). In the majority of cases, apple allergic patients exhibit OAS; however, there have also been reported instances of severe reactions following apple consumption. In our cohort, nearly half of the subjects were sensitized without allergic symptoms with apple. Among individuals presenting with allergic symptoms, 14 out of 21 (66.7%) exclusively experienced OAS, while the remaining 7 cases manifested systemic reactions. Among them, 6 out of 7 individuals exhibited cutaneous symptoms, while respiratory symptoms were observed in 4 out of 7 cases. Notably, only one individual presented with gastrointestinal symptoms. Importantly, none of the subjects experienced anaphylaxis. This symptom pattern exhibits similarities to regions with similar latitudes, such as northern Europe, but quite opposite from that of southern Europe (16, 18). In North and Central Europe, sensitization to apple is caused mainly by cross-reactive birch pollen, whereas in the Mediterranean area of Europe, apple allergy is mostly associated with allergies to peach (13).

Revealing the profiles of apple allergen components by CRD may serve as an indication of associated symptoms. Apple allergy in northern and central Europe was mild and related to Mal d 1 and Bet v 1 sensitization, whereas in southern Europe, such as Spain, apple allergy was frequently severe and related to peach allergy and sensitization to Mal d 3 (18). However, the sensitization profiles in northern China, particularly in regions where birch and mugwort are prevalent, may exhibit variations compared to those observed in northern Europe despite their similar latitudes. In a study conducted on Mediterranean patients, the recognition rates of Mal d 1, Mal d 2, Mal d 3, and Mal d 4 were found to be 27, 5, 37, and 30%, respectively (16). In our study, 85% of the subjects demonstrated sensitization to Mal d 1, while 20% exhibited sensitization to Mal d 3. These findings align with those observed in northern European regions but diverge significantly from the higher rate of sensitization to Mal d 3 reported in southern European areas.

PR-10 proteins have been identified as allergens in most *Rosaceae* fruits (7, 30). The apple allergy arises later as a result of the cross-reactivity between Bet v 1 and Mal d 1 in birch rich areas (13, 18, 31, 32). Typically, PR-10s are easily degraded, being known to induce mild allergic reactions, often limited to the oral cavity. In central and northern European countries such as the Netherlands, Austria, and parts of Italy, apple allergy typically manifests as mild symptoms and is commonly associated with birch pollen allergy, exhibiting a high sensitization rate to Mal d 1. The Japanese study yielded a similar finding, revealing a specific IgE positive rate of 92.3% against Mal d 1 (12). In a study of Korea, of 34 patients with apple allergy, 28 (82.4%) were positive for Mal d 1-specific IgE (33). In our study cohort, 85.0% tested positive for sIgE against Mal d 1, a prevalence similar to that observed in Northern Europe, Japan, and South Korea but higher than southern Europe. We also found that subjects with a higher level of Mal d 1 sIgE were more likely to suffer from apple allergy, mostly experiencing local symptoms or slightly generalized symptoms. Our study demonstrated that the AUC of Mal d 1 for differentiating apple allergy from tolerance was determined to be 0.902, with a sensitivity of 93.8% and specificity of 75.0% which was more effective than Bet v 1 (AUC 0.799) and birch (AUC 0.747). The cut-off value of Bet v 1 and Mal d 1 to discriminate between apple allergy and tolerance was calculated, being 6.84 kUA/L and 1.61 kUA/L in our cohort, and being 8.21 kUA/L and 5.3 kUA/L in Spain (16). However, Mal d 1 could not differ local or generalized symptoms to apple in our cohort which was similar to the result of southern Europe (16). Furthermore, the concentration of Mal d 1 in apple may exhibit a time-dependent increase (18, 34). A significant increase in the Mal d 1 content during storage was observed by Kaeswurm J et al. (35). In one of our patients, the absence of symptoms was observed upon consumption of fresh apples, whereas symptoms were reported following the consumption of stored apples.

The nsLTPs exhibit remarkable stability throughout the food processing procedure, and individuals with IgE antibodies against nsLTPs manifest severe allergic reactions to specific food allergens. The presence of LTP in apple, namely Mal d 3, elicited a positive response in approximately 28% of the participants reported firstly by *Pastorello* et al. (23). In the majority of studies conducted beyond the Mediterranean region, sensitization to LTPs appears to hold limited significance, often being categorized as minor allergens (17, 18, 36). Fernandez-Rivas *et al* (18) showed that sensitization to Mal d 3 was a risk factor for having systemic reactions for apple allergy. However, it depends on regions. In contrast to the occurrence of severe reactions, such as anaphylaxis, observed in cases of Mal d 3 sensitization in southern Europe, symptoms in northern regions appear to be limited to mild or moderate levels. A comparative study conducted in the Netherlands investigated patients with anaphylactic and mild reactions to apple, revealing that both groups exhibited sensitization to PR10-proteins, while only 1/7 of the mild reaction group and none of the anaphylactic reaction group demonstrated sensitization to LTP (19). To the best of our knowledge, no study in China explored the clinical significance of Mal d 3 to date. In our study, eight subjects were sensitized to Mal d 3, out of which only three exhibited apple allergic symptoms such as OAS or mild generalized symptoms. The findings suggest that in our cohort, similar to the Netherlands, LTPs does not serve as an indicative systemic allergic marker. IgE reactivity to LTP was correlated with a lower frequency of severe reactions when the patients were co-sensitized to profilin or both profilin and Bet v 1-like protein. Exclusive sensitization to Mal d 3 was not observed in any of the patients included in our study, which may account for the absence of severe reactions to apple allergy among them. This condition may be attributed to the hypothesis that co-sensitization to unrelated allergens, such as PR-10s or profilins, exhibits a weaker capacity to stimulate mast cells compared to mono-sensitization to LTP itself (18). Therefore, it is proposed that co-sensitization to profilin, PR-10s, in addition to LTPs, may exert a “protective” influence on the manifestation of LTP allergy (37).

Additionally, all subjects included in our study who exhibited sensitization to Mal d 3 also demonstrated concurrent sensitization to mugwort and presented allergic reactions toward nuts or legumes, predominantly displaying systemic symptoms. The prevalence of mugwort allergy was high in northern China, attributed to the extensive grassland coverage, whereas it exhibited lower incidence in Europe where ragweed predominated (38, 39) The disparity can be ascribed to the distinctive molecular profiles exhibited by mugwort and ragweed. Art v 3, the homologous protein of LTPs, has been identified as the major allergen in mugwort, whereas it is minor ragweed allergen component (39, 40). Conversely, Amb a 1, minor in mugwort, serves as the major allergen in ragweed. Consequently, the prevalence of mugwort-related food allergies such as peach and nut allergies is higher in northern China compared to northern Europe, Japan, and South Korea. Studies of peach allergy in northern China have recognized LTP as a primary sensitizer for severe cases (41–45). Hence, it can be postulated that the sensitization to Mal d 3 in northern China might be attributed to the homology between other LTPs, such as Pru p 3 in peach and Jug r 3 in walnut. These LTPs could potentially elicit severe systemic reactions toward nuts and legumes rather than apples.

Profilins have been characterized as allergens in most *Rosaceae* fruits, almond (Pru du 4), peach (Pru p 4), cherry (Pru av. 4), plum (Pru d 4), apple (Mal d 4), pear (Pyr c 4) and strawberry (Fra a 4) (7). Profilins are also implicated in PFAS, wherein individuals with fruit allergies experience mild oral symptoms due to co-sensitization to pollens (46). Sensitization to Mal d 4 primarily occurs with a minor role in apple allergy and exhibits strong cross-reactivity with Bet v 2. A study found that 38.06% of cases were positive to profilins only, with no differences between patients with OAS and systemic symptoms (22.85 and 15.21% of cases respectively) (16). Mono-sensitized to profilin in apple may not indicate allergic symptoms. In our study, the levels of Bet v 2 mono-sensitization ranged from 2.83 to 54.1 kUA/L in four patients, with only one patient experiencing cutaneous symptoms. The level of Bet v 1 sIgE in this patient was determined to be 0.33kUA/L, leading us to hypothesize that it may contribute to the observed phenomenon rather than profilin sensitization.

It’s worth noting that there are different isoforms of Mal d proteins which may exhibit varying IgE reactivity to different isoforms, but might not manifest symptoms despite a general allergy to Mal d (47). Further study may focus on different isoforms of Mal d 1, Mal d 3 to explore the variation of sensitization.

### 4.1 Limitations

The study has several limitations. Firstly, the present study included a relatively small sample size of individuals with apple allergy. Secondly, we were unable to analyze Mal d 2 due to the unavailability of reagents. However, previous studies have confirmed that Mal d 2 is not a major allergen in apples (sensitized rate approximately 5% (16, 18)). Therefore, the exclusion of Mal d 2 analysis in this study may not impact its clinical utility. Furthermore, the lack of a double-blind placebo-controlled apple challenge test may have hindered our ability to accurately determine allergic reactions to apples.

### 4.2 Strengths

This study possesses several notable strengths. Firstly, we conducted the pioneering analysis of allergen components in Chinese apple allergy subjects. Secondly, we successfully validated the diagnostic efficacy of Mal d 1 in determining apple allergy or tolerance. Thirdly, we observed regional variations in the significance of Mal d 3 (nsLTP) and established its association with mugwort allergy in northern China. The findings provide valuable insights into the characteristics of apple allergy in China and contribute to a better understanding of apple allergy on a global scale.

### 4.3 Conclusion

In conclusion, our findings have revealed a relatively high sensitization rate of Mal d 1, which is comparable to the rates observed in northern Europe and Japan. The sIgE level of Mal d 1 was found to be reliable and superior for diagnosing apple allergy compared to apple sIgE. However, it should be noted that sensitization to Mal d 3 does not necessarily indicate a severe allergic reaction to apples in northern China; instead, it may indicate mugwort sensitization and an increased risk of peach, nuts or legumes allergy.

## Data availability statement

The raw data supporting the conclusions of this article will be made available by the authors, without undue reservation.

## Ethics statement

The studies involving humans were approved by Beijing Shijitan hospital, capital medical university. The studies were conducted in accordance with the local legislation and institutional requirements. Written informed consent for participation in this study was provided by the participants' legal guardians/next of kin.

## Author contributions

XiW: Conceptualization, Data curation, Formal analysis, Funding acquisition, Methodology, Validation, Visualization, Writing – original draft. LC: Data curation, Formal analysis, Writing – review & editing, Investigation. TL: Writing – review & editing, Data curation, Methodology. HW: Writing – review & editing, Conceptualization, Resources, Supervision. XuW: Conceptualization, Supervision, Writing – review & editing, Funding acquisition, Validation.
